# Pilot Study on Image Quality and Radiation Dose of CT Colonography with Adaptive Iterative Dose Reduction Three-Dimensional

**DOI:** 10.1371/journal.pone.0117116

**Published:** 2015-01-30

**Authors:** Hesong Shen, Dan Liang, Mingyue Luo, Chaijie Duan, Wenli Cai, Shanshan Zhu, Jianping Qiu, Wenru Li

**Affiliations:** 1 Department of Radiology, The Sixth Affiliated Hospital of Sun Yat-sen University, Guangzhou, Guangdong, China; 2 Research Center of Biomedical Engineering, Graduate School at Shenzhen, Tsinghua University, Shenzhen, Guangdong, China; 3 Department of Radiology, Massachusetts General Hospital and Harvard Medical School, Boston, Massachusetts, United States of America; University of Nebraska Medical Center, UNITED STATES

## Abstract

**Objective:**

To investigate image quality and radiation dose of CT colonography (CTC) with adaptive iterative dose reduction three-dimensional (AIDR3D).

**Methods:**

Ten segments of porcine colon phantom were collected, and 30 pedunculate polyps with diameters ranging from 1 to 15 mm were simulated on each segment. Image data were acquired with tube voltage of 120 kVp, and current doses of 10 mAs, 20 mAs, 30 mAs, 40 mAs, 50 mAs, respectively. CTC images were reconstructed using filtered back projection (FBP) and AIDR3D. Two radiologists blindly evaluated image quality. Quantitative evaluation of image quality included image noise, signal-to-noise ratio (SNR), and contrast-to-noise ratio (CNR). Qualitative image quality was evaluated with a five-score scale. Radiation dose was calculated based on dose-length product. Ten volunteers were examined supine 50 mAs with FBP and prone 20 mAs with AIDR3D, and image qualities were assessed. Paired t test was performed for statistical analysis.

**Results:**

For 20 mAs with AIDR3D and 50 mAs with FBP, image noise, SNRs and CNRs were (16.4 ± 1.6) HU vs. (16.8 ± 2.6) HU, 1.9 ± 0.2 vs. 1.9 ± 0.4, and 62.3 ± 6.8 vs. 62.0 ± 6.2, respectively; qualitative image quality scores were 4.1 and 4.3, respectively; their differences were all not statistically significant. Compared with 50 mAs with FBP, radiation dose (1.62 mSv) of 20 mAs with AIDR3D was decreased by 60.0%. There was no statistically significant difference in image noise, SNRs, CNRs and qualitative image quality scores between prone 20 mAs with AIDR3D and supine 50 mAs with FBP in 10 volunteers, the former reduced radiation dose by 61.1%.

**Conclusion:**

Image quality of CTC using 20 mAs with AIDR3D could be comparable to standard 50 mAs with FBP, radiation dose of the former reduced by about 60.0% and was only 1.62 mSv.

## Introduction

Colorectal cancer is the fourth leading cause of cancer death in China, and is usually diagnosed at an advanced stage with a subsequent poor prognosis [1]. Early detection and removal of the precursor lesion significantly reduces the incidence and mortality associated with this neoplasm. CT colonography (CTC) is likely to become a new imaging technique for colorectal cancer screening [2–4]. A potential obstacle to mass screening CTC is the concern about radiation exposure. Researchers continuously optimize CT scan protocols to reduce radiation dose while maintaining diagnostic image quality, with manufacturers also improving hardware and software [5].

Methods for decreasing radiation dose include reducing tube current and peak voltage, increasing gantry rotation and table speed. But these methods produce higher image noise, leading to poor image quality [5]. Radiation dose of CTC has been substantially reduced through technical advancements such as new iterative reconstruction without compromising of image quality for diagnostic needs [6–8]. Adaptive iterative dose reduction three-dimensional (AIDR3D) is a recently introduced iterative reconstruction used on 640-slice CT, it can lower image noise and substantial reduce radiation dose when compared with standard filtered back projection (FBP) while maintaining image quality that meets the diagnostic needs of related lesions [9, 10].

To the best of our knowledge, there is no report about image quality and radiation dose of CTC using AIDR3D. Thus, the purpose of this pilot study was to investigate image quality and radiation dose of CTC with AIDR3D.

## Materials and Methods

### Ethics statement

We obtained ethical approval from Ethics Committee of the sixth Affiliated Hospital of Sun Yat-sen University in China. The ethical approval covered 10 segments of 50 cm long freshly isolated porcine colons and 10 healthy volunteers. The possible hazard of radiation exposure was explained to every volunteer. All volunteers provided written informed consent to participate in this study.

### Establishment of colon polyp phantom

Ten segments of 50 cm long freshly isolated porcine colons were collected in this in vitro experiment. A local abattoir provided the porcine colons (from Landrace pigs previously slaughtered for human consumption). The approximate age of pigs was 1 years old. By turning the mucosal surfaces outwards, nodules to simulate pedunculate polyps were created by lifting the mucosa and underlying tissue with toothless forceps, and tying at the root with gauge 4 suture. The diameters of these polyps ranged from 1 to 15 mm, and the height of each polyp was larger than half of its respective diameter. All the polyps were placed randomly on the mucosal surface. In total, thirty polyps were created for each colon segment, with ten for each group of 1–5 mm, 6–10 mm and 11–15 mm. The mucosal surfaces were then turned inwards to restore the original wall structure. With one end tied, each colon was inflated with JS-628F CTC inflator (Guangzhou Jinjian Technology Co., Ltd., Guangzhou, China) using a plastic anal canal. After complete inflation, the entire colon phantom was sealed by typing the other end. Extra caution was taken to prevent perforation. The inflated colon phantom was submerged and fixed in a plastic box filled with 40 liters of diatrizoate meglumine with a CT attenuation of 40 Hounsfield Unit (HU).

### CT image data acquisition

The ten colon segments were scanned on a 640-slice CT scanner (Acquilion ONE; Toshiba Medical System, Tochiki-ken, Japan) with tube voltage of 120 kVp, and current doses of 10 mAs, 20 mAs, 30 mAs, 40 mAs, 50 mAs, respectively. The imaging parameters are 500 ms rotation time, 0.5 mm × 640 slice collimation, 0.875 pitch, 512 × 512 matrix, and 400 mm field of view. The original image data were transferred to an image postprocessing workstation (Vitrea, Version 6.2; Toshiba Medical System, Tochiki-ken, Japan) via picture archiving and communication system.

### Image reconstruction

In the image postprocessing workstation, the original image data of the colon phantoms were reconstructed with FBP and AIDR3D (strong mode), and with soft tissue reconstruction function being FC43, reconstruction thickness of 1 mm, and reconstruction interval of 1 mm. Combining the five current doses and two reconstructions, a total of ten reconstruction datasets were generated, namely 10 mAs with FBP, 10 mAs with AIDR3D, 20 mAs with FBP, 20 mAs with AIDR3D, 30 mAs with FBP, 30 mAs with AIDR3D, 40 mAs with FBP, 40 mAs with AIDR3D, 50 mAs with FBP, and 50 mAs with AIDR3D. The two-dimensional transverse images and three-dimensional endoluminal images were obtained after postprocessing the ten reconstruction datasets with CTC software (Vitrea Advanced Colon, Toshiba Medical System, Tochiki-ken, Japan).

### Quantitative evaluation of image quality

All scanner demographic data were removed from CTC images. Two experienced radiologists, who were blind to current doses and image reconstructions, independently evaluated the ten sets of CTC images for image noise, signal-to-noise ratios (SNRs), and contrast-to-noise ratios (CNRs). In case of discrepant evaluation, another senior radiologist would be involved for discussion to facilitate a consensus. CT attenuations of colonic polyps, of luminal air and of room air around the plastic boxes were respectively measured. When measuring CT attenuation of colonic polyp, a region of interest (ROI) was chosen inside the polyp as large as possible without exceeding the boundary of polyp. While measuring those of the luminal air and room air around plastic box, circular ROI with diameter of 10 mm was used. Three different locations were chosen for ROI measurement of each material. The final CT attenuation was calculated by averaging three ROI measurements in each material. Image noise was represented by the standard deviation of CT attenuation of room air around the plastic box. SNR and CNR were calculated as below: SNR = (CT attenuation of colonic polyp)/(image noise), CNR = (CT attenuation of colonic polyp﹣CT attenuation of luminal air)/(image noise).

### Qualitative evaluation of image quality

The aforementioned two radiologists performed the qualitative evaluation of CTC image quality in the same blinded manner. According to the criteria for qualitative evaluation of image quality in CTC reported by Yoon MA et al [9], overall qualitative image qualities were classified into five scales: score 5, excellent, clear image with barely any artifacts; score 4, good, clear image with few artifacts; score 3, moderate, mild artifacts, not affecting the diagnosis; score 2, fair, moderate artifacts that would affect diagnosis; and score 1, poor, severe artifacts making a diagnosis impossible. Images with a quality score of 3 to 5 could be used for diagnosis, whereas images with a quality score of 1 or 2 could not be used for diagnostic purpose.

### Calculation of radiation dose

Dose-length product in each scanning reported on the CT scanner was recorded. Radiation dose was calculated as follows: radiation dose = (dose-length product) × (conversion factor), where conversion factor refers to conversion factor of different body parts. Conversion factor value for abdomen is 0.015 mSv×mGy×cm^–1^ [11].

### Statistical analysis　

Continuous data were expressed as means with standard deviations unless otherwise specified. The statistical analysis was performed with SPSS software (version 16.0, SPSS Inc). The quantitative and qualitative evaluation of CTC images were compared using the paired Student *t* test. A *P* value of 0.05 or less was considered to be statistically significant.

### Clinical assessment

Ten healthy volunteers aged 22 to 66 years were recruited, including 6 men and 4 women. CTC image acquisition was performed following standard intestinal cleansing preparation, in the order of supine scanning at 50 mAs with FBP, and prone scanning at 20 mAs with AIDR3D. For each volunteer, CT attenuations of left psoas muscle at L5 vertebral level, luminal air of the colon, and room air around the volunteer were measured, respectively. CT attenuation of luminal air was calculated by averaging those measurements in rectosigmoid junction, splenic flexure of the colon and cecum. The CT attenuations of polyp and room air were measured in the same manner as in phantom study. Image noise was defined as the standard deviation of CT attenuation of room air around the volunteer. SNR and CNR were defined as follows: SNR = (CT attenuation of psoas muscle at L5 vertebral level)/(image noise), CNR = (CT attenuation of psoas muscle at L5 vertebral level﹣CT attenuation of luminal air)/(image noise). Three representative sites, namely rectosigmoid junction, splenic flexure of the colon and ileocecal valve, were selected for qualitative evaluation of CTC images, and the mean value was used as the overall qualitative image quality. Other methods were the same as those in phantom study.

## Results

### Quantitative evaluation of image quality

Results of quantitative evaluation of CTC image quality are summarized in Table 1. At the same level of radiation dose, AIDR3D resulted in less image noise, higher SNRs and CNRs, compared with FBP. For the sets of 20 mAs with AIDR3D and 50 mAs with FBP, image noise were (16.4 ± 1.6) HU and (16.8 ± 2.6) HU, respectively, without statistically significant difference (*P* = 0.063); SNRs were 1.9 ± 0.2 and 1.9 ± 0.4, without statistically significant difference (*P* = 0.129); and CNRs were 62.3 ± 6.8 and 62.0 ± 6. 2, respectively, without statistically significant difference (*P* = 0.064). In the set of 10 mAs with AIDR3D, image noise was (21.2 ± 2.5) HU, being significantly higher than that of 50 mAs with FBP ((16.8 ± 2.6) HU, *P* = 0.002); SNR was 1.7 ± 0.3, being significantly lower than that of 50 mAs with FBP (1.9 ± 0.4, *P* = 0.000); and CNR was 48.4 ± 5.7, being significantly lower than that of 50 mAs with FBP (62.0 ± 6.2, *P* = 0.000).

**Table 1:** Quantitative evaluation of CTC image quality with FBP and AIDR3D.

Evaluation item	10 mAs with FBP	10 mAs with AIDR3D	20 mAs with FBP	20 mAs with AIDR3D	30 mAs with FBP	30 mAs with AIDR3D	40 mAs with FBP	40 mAs with AIDR3D	50 mAs with FBP	50 mAs with AIDR3D
Noise	48.0±3.1	21.2±2.5	32.5±3.2	16.4±1.6	25.2±2.6	14.0±1.8	23.1±3.1	13.0±2.0	16.8±2.6	11.8±1.4
SNR	0.9±0.1	1.7±0.3	1.3±0.2	1.9±0.2	1.4±0.2	2.1±0.4	1.6±0.4	2.7±0.3	1.9±0.4	3.0±0.4
CNR	21.4±1.3	48.4±5.7	31.8±3.4	62.3±6.8	40.9±4.6	73.2±9.8	54.7±5.9	79.7±11.7	62.0±6.2	85.8±10.9

### Qualitative evaluation of image quality

**Fig 1A-J:**
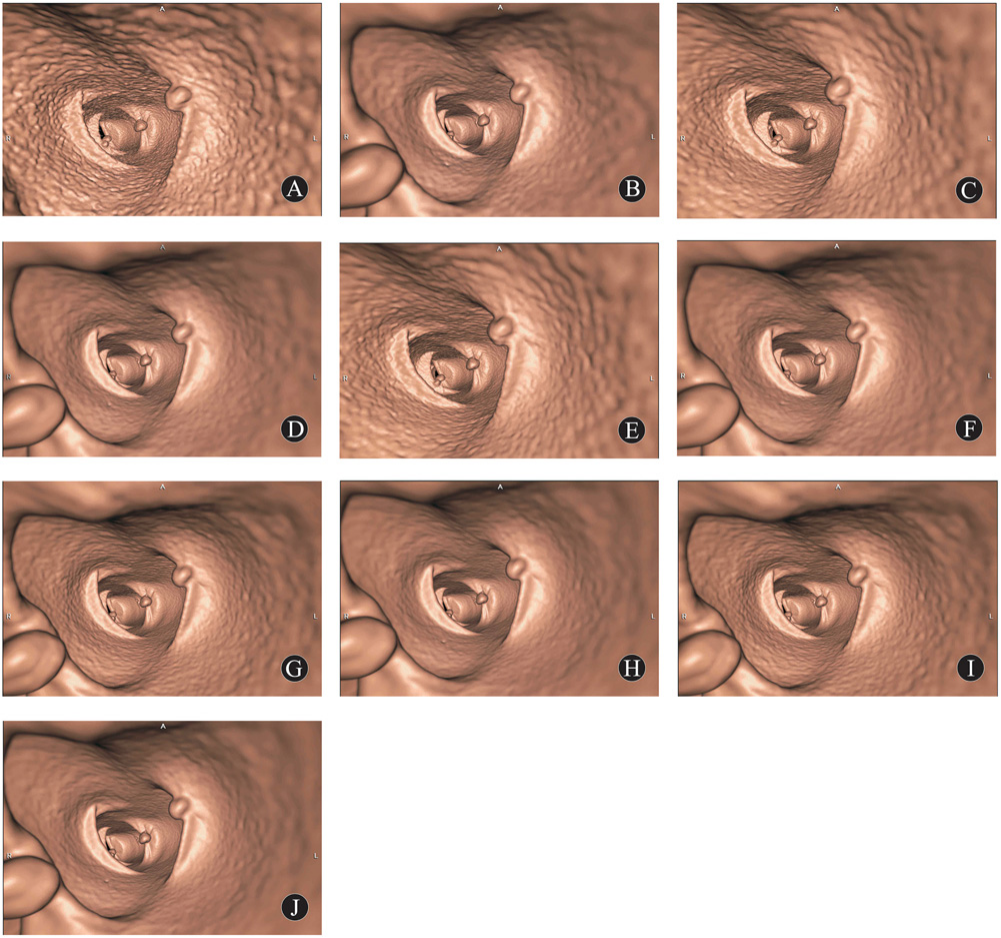
Comparison of qualitative CTC images of porcine colons using 5 current doses with 2 reconstructions. CTC images of 10 mAs with FBP, 10 mAs with AIDR3D, 20 mAs with FBP, 20 mAs with AIDR3D, 30 mAs with FBP, 30 mAs with AIDR3D, 40 mAs with FBP, 40 mAs with AIDR3D, 50 mAs with FBP, 50 mAs with AIDR3D, respectively; their qualitative evaluation scores are 1.9, 2.8, 2.5, 4.1, 3.7, 4.5, 4.1, 4.7, 4.3, 4.8, respectively.

Qualitative evaluation scores of CTC images are shown in Table 2. At the same level of radiation dose, AIDR3D yielded better qualitative evaluation scores than did FBP. The scores of image quality in the sets of 10 mAs with FBP, 10 mAs with AIDR3D, and 20 mAs with FBP were 1.9, 2.8 and 2.5, respectively, suggesting that the images could not be used for diagnostic purpose. The scores in the sets of 20 mAs with AIDR3D and 50 mAs with FBP were 4.1 and 4.3, respectively, without statistically significant difference (*P* = 0.121). Score of images in the set of 10 mAs with AIDR3D was 2.8, being significantly lower than that in 50 mAs with FBP (4.3, *P* = 0.000) (Fig. 1A-J).

**Table 2:** Qualitative evaluation of CTC image quality with FBP and AIDR3D.

Reconstruction technique	10 mAs	20 mAs	30 mAs	40 mAs	50 mAs
FBP	1.9	2.5	3.7	4.1	4.3
AIDR3D	2.8	4.1	4.5	4.7	4.8

### Radiation dose

Dose-length products for current doses of 10 mAs, 20 mAs, 30 mAs, 40 mAs and 50 mAs were 53.9 mGy.cm, 107.7 mGy.cm, 161.6 mGy.cm, 215.4 mGy.cm and 269.3 mGy.cm, respectively. Radiation doses were 0.81 mSv, 1.62 mSv, 2.42 mSv, 3.23 mSv and 4.04 mSv, respectively. Compared with 50 mAs with FBP, radiation dose for 20 mAs with AIDR3D was lowered by 60.0%, their difference had statistical significance (*P*﹤0.001).

### Clinical assessment

Image noise of prone 20 mAs with AIDR3D and supine 50 mAs with FBP for the ten human CTC images were (26.4 ± 1.9) HU and (26.7 ± 2.8) HU, respectively; SNRs were 2.4 ± 1.0 and 2.3 ± 0.6, respectively; and CNRs were 100.1 ± 8.0 and 98.4 ± 6.6, respectively; their differences were all not statistically significant (all *P*﹥0.05). Qualitative evaluation scores of CTC image quality were 3.9 and 4.0, respectively, without statistically significant difference (*P*﹥0.05). Radiation doses were 1.4 mSv and 3.6 mSv, respectively, with a reduction of 61.1% between prone 20 mAs with AIDR3D and supine 50 mAs with FBP, their difference was statistically significant (*P*﹤0.05).

## Discussion

CTC has been shown to be a new effective tool for colorectal cancer screening, due to its non-invasiveness and high sensitivity for polyp and neoplasia detection [12–16]. However, its hazard of ionizing radiation is a major concern to the public [17–19]. CTC should follow the principle of maintaining radiation dose as low as reasonably achievable (ALARA), that is, to decrease radiation dose to the minimum level without compromising image quality [20–28]. It is crucial to balance the relationship between radiation dose and image quality that meets diagnostic needs [29]. Main methods for decreasing radiation dose include reducing current dose (mAs), reducing tube voltage, applying automatic tube current modulation, and using iterative reconstruction [8, 30]. There is a direct linear relationship between mAs and radiation dose. The most straightforward approach to decrease radiation dose is to reduce mAs. Nevertheless, reducing mAs will increase image noise and lower image quality. Reducing mAs alone can only decrease radiation dose by a limited extent [31]. Therefore, focus of research on CTC has developed from only reduction of mAs to combined use of new image reconstruction without compromising image quality for diagnostic needs.

Currently, CTC uses a standard current dose of 50 mAs. FBP is a conventional CT image reconstruction [32]. Fast as it is, FBP can not distinguish basic components in image data acquisition, and brings noises into reconstructed images because of ignoring contamination in image data acquisition process by quantum noises and electronic noises, thus affecting image quality and lesion detection. In an experimental study with porcine colon simulated polyps using 10 mAs with FBP by Branschofsky M et al [22], polyps were deformed on images, and image quality was too poor to be used for diagnosis. In our study, image noise, SNR and CNR of CTC images using 50 mAs with FBP were (16.8 ± 2.6) HU, 1.9 ± 0.4 and 60.2 ± 6.2, respectively; qualitative evaluation of image quality was scored 4.3, suggesting that the images could be used for diagnosis. Although the sensitivity was 100% for simulated polyps of 1–5 mm, 6–10 mm and 11–15 mm, its radiation dose was up to 4.04 mSv.

AIDR3D is a newly introduced three-dimensional adaptive iterative dose reduction image reconstruction developed for 640-slice CT scanner by Toshiba Medical System. Through the establishment of a noise model, using FBP image as original building blocks, and utilizing statistical and algebraic iterative algorithm for X-ray noise and electronic noise filtering, it can adaptively balance between noise suppression and quality details by repeated spatial filtering for noise suppression until a pre-defined target is reached. It is expected to break the limit of FBP by reducing image noise associated with low dose scanning and suppressing image artifacts, thereby improving image quality, which is conducive to detection of lesion [33–36]. The ratio of AIDR3D is a mixed ratio of original FBP image and noise free AIDR3D image generated through a mathematical model. A 0% AIDR3D image is FBP image, and a 100% AIDR3D image is noise free AIDR3D image. The higher the percentage, the better the image quality despite longer reconstruction time. In a study using automatic exposure mAs and standard AIDR3D in 640-slice CT coronary angiography, Roo RE et al [37] found that compared with FBP, standard AIDR3D reduced image noise by 38.6%, increased SNR by 63.0% and CNR by 61.5%. Up to now, the application of AIDR3D at 640-slice CT scanner has not been reported for CTC.

In this study, strong AIDR3D was used. By combining strong AIDR3D and FBP, we achieved a balance between improved image quality and shorter reconstruction time. Compared with 50 mAs with FBP, image noise, SNR and CNR of 50 mAs with AIDR3D were (11.8 ± 1.4) HU (reduced by 29.7%), 3.0 ± 0.4 (increased by 57.8%) and 85.8 ± 10.9 (increased by 38.4%), respectively. There was no statistically significant difference in image noise, SNR, CNR and qualitative image quality evaluation score between 20 mAs with AIDR3D and 50 mAs with FBP, though the former reduced radiation dose by 60.0%. However, the lower current dose, that is 10 mAs with AIDR3D, had higher image noise, lower SNR and CNR as well as qualitative image quality evaluation score, compared with 50 mAs with FBP, with statistically significant difference. Based on the phantom study results, ten volunteers received supine 50 mAs with FBP and prone 20 mAs with AIDR3D CTC examinations. This preliminary clinical study showed no statistically significant difference in image noise, SNR, CNR and qualitative image quality evaluation score between the two examinations, though the latter reduced radiation dose by 61.1%, which was consistent with the results in phantom study.

Our study is limited with respect to ideal colonic condition, single polyp morphology, small number of subjects, and one single CT scanner. First, compared with clinical cases, *in vitro* model establishment of porcine colonic polyps was carried out under idealized condition. The intestinal structure and major abdominal blood vessels in human beings were not simulated, and impact by intestinal peristalsis and pulsation of major abdominal blood vessels was not considered. Second, only pedunculate polyps were simulated in the study, no flat and sessile polyps were included, making the model of polyps monotonous in shape. These two types of polyps are sometimes difficult to be distinguished from intestinal mucosal folds, so that polyp detectability in this experimental study may have been higher than that in clinical examinations. Third, there were only 10 volunteers in the clinical assessment. Due to the small sample size, clinical study was merely preliminary. Fourth, we only included one single CT scanner (640-slice CT scanner, Acquilion ONE; Toshiba Medical System), its iterative reconstruction may not be the same as those of other CT scanners by different vendors. Therefore, further studies with a larger number of clinical subjects are required to further evaluate and confirm possible clinical application of CTC using 20 mAs with AIDR3D.

In conclusion, our pilot phantom and preliminary clinical studies show that image quality of CTC using 20 mAs with AIDR3D could be comparable to currently standard 50 mAs with FBP, radiation dose of the former reduced by about 60.0% and was only 1.62 mSv.
